# Subfertility and Risk of Testicular Cancer in the EPSAM Case-Control Study

**DOI:** 10.1371/journal.pone.0169174

**Published:** 2016-12-30

**Authors:** Chiara Grasso, Daniela Zugna, Valentina Fiano, Nena Robles Rodriguez, Milena Maule, Anna Gillio-Tos, Libero Ciuffreda, Patrizia Lista, Nereo Segnan, Franco Merletti, Lorenzo Richiardi

**Affiliations:** 1 Cancer Epidemiology Unit-CeRMS, Department of Medical Sciences, University of Turin and CPO Piedmont, Turin, Italy; 2 Medical Oncology Division 1, University Hospital “Città della Salute e della Scienza”, Turin, Italy; 3 Department of Cancer Screening and Unit of Cancer Epidemiology, WHO Collaborative Center for Cancer Early Diagnosis and Screening, CPO Piedmont and University Hospital “Città della Salute e della Scienza”, Turin, Italy; Ruhr-Universitat Bochum, GERMANY

## Abstract

**Background/objectives:**

It has been suggested that subfertility and testicular cancer share genetic and environmental risk factors. We studied both subfertility and the strongest known testicular cancer susceptibility gene, the c-KIT ligand (KITLG), whose pathway is involved in spermatogenesis.

**Methods:**

The EPSAM case-control study is comprised of testicular cancer patients from the Province of Turin, Italy, diagnosed between 1997 and 2008. The present analysis included 245 cases and 436 controls from EPSAM, who were aged 20 years or older at diagnosis/recruitment. The EPSAM questionnaire collected information on factors such as number of children, age at first attempt to conceive, duration of attempt to conceive, use of assisted reproduction techniques, physician-assigned diagnosis of infertility, number of siblings, and self-reported cryptorchidism. Genotyping of the KITLG single nucleotide polymorphism (SNP) rs995030 was performed on the saliva samples of 202 cases and 329 controls.

**Results:**

Testicular cancer was associated with the number of children fathered 5 years before diagnosis (odds ratio (OR) per additional child: 0.78, 95% confidence interval (CI): 0.58–1.04) and sibship size (OR per additional sibling: 0.76, 95% CI: 0.66–0.88). When considering the reproductive history until 1 year before diagnosis, attempting to conceive for at least 12 months or fathering a child using assisted reproduction techniques was not associated with the risk of testicular cancer, nor was age at first attempt to conceive or physician-assigned diagnosis of infertility. The SNP rs995030 was strongly associated with risk of testicular cancer (per allele OR: 1.83; 95%CI: 1.26–2.64), but it did not modify the association between number of children and the risk of testicular cancer.

**Conclusion:**

This study supports the repeatedly reported inverse association between number of children and risk of testicular cancer, but it does not find evidence of an association for other indicators of subfertility.

## Introduction

Testicular cancer is a relatively rare malignancy and occurs most commonly in young and middle-aged men [1, 2]. Although the incidence of testicular cancer is low worldwide, over the last decades it has increased by 3–4% per year in several populations [3]. There are few well-established risk factors for testicular cancer, including cryptorchidism, contralateral testicular cancer, family history of testicular cancer, and height [4]. Ninety-five percent of testicular cancers are germ cell tumours, which can be subcategorized into seminomas and nonseminomas.

There is a large amount of literature on the association between subfertility and the risk of testicular cancer. Large register-based studies in the Nordic countries found that men with testicular cancer fathered fewer children before their diagnosis than did their corresponding control populations [5–7], and this association has been replicated in other case-control studies [8–10]. Furthermore, men with testicular cancer tend to have fewer siblings, which might suggest a reduced fertility in their parents [11–13].

The associations between the risk of testicular cancer and other questionnaire-based indicators of infertility (e.g. difficulty impregnating a partner or reported diagnosis of infertility) and clinical measures of infertility (e.g. sperm count and motility, serum Inhibin B, FSH, and testosterone) are less consistent and more difficult to investigate than the number of children fathered, due to problems of reverse causation, measurement errors, and recall and detection bias. Cohort studies of men evaluated for infertility [14–19] have found an increased risk of testicular cancer among those with abnormal semen analyses or male-factor infertility. These studies have the strength of prospective measurements of fertility, often using semen analysis, but they may be affected by other sources of bias, including confounding by cryptorchidism, and detection bias. Furthermore, couples seeking medical advice for fertility problems are a selected sample, whose data might not be comparable with population-based external cancer incidence data.

Malignancies of the testis have an important inherited component: brothers and sons of testicular cancer patients have an 8–10 and 4–6 fold increased risk of testicular cancer, respectively [20].

Genome-wide association studies (GWAS) have detected more than twenty loci that are associated with the risk of testicular cancer [21–28]. Notably, two independent GWAS found that allele variations in KITLG (the unique ligand for the receptor tyrosine kinase KIT) were the strongest genetic risk factors for testicular germ cell tumors [21, 22]. These studies showed that the KITLG single nucleotide polymorphism (SNP) rs995030 was associated with an at least 2-fold increased risk of testicular cancer. The other identified SNPs were in strong linkage disequilibrium with each other and with rs995030 (r^2^>0.8), which may thus serve as a TagSNP. Apart from these SNPs, other genetic alterations of the KIT/KITLG pathway have been linked to an increased susceptibility to testicular germ cell tumors [29–33].

The KIT–KITLG system is also crucial for germ cell survival, proliferation, motility, and migration [34], and its alterations may impair fertility. Indeed, mutations [35, 36], complete or partial deletions of KIT or KITLG sequences [37, 38] and altered expression of either genes have been reported to be involved in the disruption of normal germ cell development [39, 40], in increased spermatocyte apoptosis [41], and in microenvironment alterations [42].

A few epidemiological studies found some empirical evidence of an association between KIT/KITLG SNPs and the risk of male infertility, although the results were not entirely consistent [43–45].

We used data from the EPSAM case-control study, carried out in the North of Italy, to estimate the role of subfertility in the aetiology of testicular cancers. In order to disentangle the possible roles of shared environmental and genetic risk factors, we also assessed interactions the KITLG SNP rs995030 may have with subfertility in its association with the risk of testicular cancer.

## Material and Methods

The EPSAM study was approved by the Ethical Committee (IRB) of the San Giovanni Battista Hospital–CTO/CRF/Maria Adelaide Hospital (Turin, Italy). All individual participants included in the study signed informed consent forms.

### The EPSAM study

The EPSAM case-control study is comprised of patients resident in the Province of Turin, Italy diagnosed with a histologically-confirmed testicular germ cell tumor between 1997 and 2008 and their controls.

Testicular germ cell cancer cases who underwent an orchiectomy (ICD-9 CM surgical procedure codes: 623–624) for testicular cancer (ICD-9 CM diagnostic code: 186) during the study period were identified in regional Hospital Discharge Registry. Those patients who were treated at the Oncology Ward of the San Giovanni Battista Hospital (the main hospital of the city of Turin) were contacted through their oncologist; patients who were treated at other hospitals were contacted through their GPs (81% of the contacted GPs with eligible patients agreed to collaborate). Histological reports of all cases were consulted to determine the subtype of the germ cell tumors (seminoma or nonseminoma). One case without histological confirmation was included in the study on the basis that 95% of testicular tumors are germ cell cancers.

Up to two population-based controls were selected per case: for each case contacted through his GP, we randomly selected two men from the list of the same GP, matched by year of birth and residence (Turin or rest of the province of Turin), and for each case contacted through the San Giovanni Battista Hospital, we contacted up to two patients frequency matched by birth year and residence among patients admitted at the same hospital for non-neoplastic diseases that were unrelated to hormonal factors, fertility status, or testicular health.

All cases and controls completed a questionnaire that collected information on age at diagnosis, exposures occurring during puberty, adolescence, and early adulthood, as well as on main risk factors for testicular cancer. This included year of birth, residence, and education level, as well as detailed information on fertility: number of children, information on each child fathered, including date of birth, age at first attempt to conceive, duration of attempts to conceive, use of assisted reproduction techniques, number of months of attempts to conceive and if they ever visiting a doctor for infertility problems, physician-assigned diagnosis of male- or female-factor infertility, number of siblings (which was used to calculate sibship size), and self-reported cryptorchidism. The response proportion was 57% among cases and 48% among controls.

Cases and controls were also asked to self-collect and mail a saliva sample using the Oragene DNA (OG-250) Collection Kit (DNA Genotek Inc., Ottawa, Canada), with a response proportion of 84% among both cases and controls.

Full details of the EPSAM study design have been described in previous publications [46, 47]. Anonymized data on fertility status and genotyping of cases and controls are available upon request to qualified researchers for the purpose of academic, non-commercial research.

### Study sample

For the purposes of this analyses, we restricted the study sample to participants born in Italy (5 cases and 6 controls excluded) and aged at least 20 years at diagnoses/recruitment (15 cases and 31 controls excluded), as it is unlikely that attempts to conceive happen before this age. This left 245 cases 436 controls in the analytical sample. Genotyping of the KITLG single nucleotide polymorphism (SNP) rs995030 was performed on the saliva samples of 202 cases and 342 controls.

### Molecular analyses

DNA was extracted from 1 ml of saliva using the Oragene Purifier solution (OG-L2P) following manufacturer's protocol. The β-globin gene fragment (268 bp) was amplified by PCR using previously published conditions [48] and analyzed in 2% agarose-gel electrophoresis. One case and 15 controls were excluded from genotyping due to DNA inadequacy.

Genotyping of the SNP rs995030 within the 3’UTR of the KITLG gene (Chromosome 12, NCBI Nucleotide Reference Sequence: NG_012098.1) was performed. Primers, designed using the PyroMark Assay Design software version 2.0 (Qiagen, Hilden, Germany) were: 5’-ACTGTGAAACTGGCACTGAATTAA-3’ (forward), 5’-BIOT-CTTGCAGAGACCAGGATAACTACA-3’ (reverse), 5’-CGTGTCTCAGACTGCAT-3’(sequencing). PCR was carried out in a final volume of 35 μl containing 20 ng of genomic DNA, 1X PCR Gold Buffer, 1.5 mM MgCl2 (Applied Biosystems, Foster City, CA), 0.8 mM dNTPs mix (Promega, Madison, WI, USA), 0.5 μmol of each amplification primer (Sigma Aldrich, St. Louis, MO, USA) and 0.05 U of AmpliTaq Gold DNA polymerase (Applied Biosystems). The PCR termic profile was: denaturation at 95°C (10 min) followed by 45 cycles of 95°C (30 s), 60°C (1 min), 72°C (1 min), and a final extension step at 72°C (10 min). Pyrosequencing analysis was performed onto a PyroMark Q24 MDx Pyrosequencing System (Qiagen).

### Statistical analyses

Information on fertility from questionnaires was used to create indicators of fertility. First, we analyzed the number of children fathered by cases and controls before diagnosis of testicular cancer (for controls a reference age was randomly assigned on the basis of the age distribution at diagnosis of the cases). We then analyzed the age at first attempt to conceive, irrespective of its success. This is a potential confounder of the association between number of children and risk of testicular cancer, because earlier age at first attempt gives more opportunities to conceive in analyses that are restricted to the period before cancer diagnosis, and thus cannot cover the entire reproductive period, and because fertility declines with age. As a more direct assessment, we also constructed a combined indicator of fertility based on the duration of attempts to conceive for men whose attempts were successful and for those whose attempts were not: (1) had children without attempting to conceive or after attempting for less than 12 months; (2) did not have children and did not attempt to conceive; (3) attempted to conceive for at least 12 months and had/did not have children or had children using assisted reproduction techniques.

When calculating the number of children fathered, we introduced lag-times before testicular cancer diagnosis (reference age for controls) of 1 year and 5 years to increase the study’s comparability with other studies. For the other indicators of fertility we used a lag-time of 1 year.

We used unconditional logistic regression to estimate odds ratios (ORs) with corresponding 95% confidence intervals (CIs) of testicular cancer, adjusting for matching variables–year of birth, residence (city of Turin, rest of the Province of Turin), method of recruitment (GPs or hospital oncologist)–as well as for age at diagnosis/reference age, educational level (junior high school, high school, university degree), and cryptorchidism. Analyses on age at first attempt to conceive were restricted to cases and controls who attempted to conceive before the diagnosis of testicular cancer or the reference age for controls.

We performed sensitivity analyses to check for possible biases in the analyses on fertility. First, we restricted the analysis to subjects who did not report cryptorchidism, and second to cases and controls aged at least 35, who have a more complete reproductive history.

For the analyses on the KITLG SNP rs995030, we first tested for the Hardy-Weinberg equilibrium (HWE) among controls. We used unconditional logistic regression to estimate the OR of testicular cancer for each genotype according to the codominant model, as well as the OR for each allele according to an additive model.

We also performed stratified analyses by histological subtype (seminoma or nonseminoma histology). Stata software version 12.0 (Stata Corporation, College Station, Texas) was used for the analyses.

## Results

Characteristics of the 245 testicular cancer cases and 436 controls included in this study are summarized in Table 1. Cases and controls had similar educational levels while cryptorchidism was, as expected, more frequent among cases. Of the 245 cases, 136 had seminoma and 108 had nonseminoma histology.

**Table 1:** Selected characteristics of cases and controls.

Characteristic	Cases (N = 245)		Controls (N = 436)	
	N	(%)	N	(%)
**Year of birth**				
<1960	47	19.2	101	23.2
1960–69	80	32.6	140	32.1
1970–79	100	40.8	165	37.8
1980+	18	7.4	30	6.9
**Method of recruitment**				
General Practitioners	163	66.5	300	68.8
Hospital	82	33.5	136	31.2
**Residence**				
City of Turin	95	38.8	189	43.3
Province of Turin	150	61.2	247	56.7
**Education level**				
Junior high school	90	36.7	154	35.4
High school	98	40.0	190	43.7
University degree	57	23.3	91	20.9
Missing	0		1	
**Cryptorchidism**^**a**^				
No	203	87.9	401	96.9
Yes	28	12.1	13	3.1
Missing	14		22	
**Histology**				
Seminoma	136	55.7	-	
Nonseminoma	108	44.2	-	
Missing	1		-	

^a^ Self-reported physician-diagnosed cryptorchidism.

As reported in Table 2, we observed an inverse association with risk of testicular cancer for number of children fathered by testicular cancer cases, although CIs were large (OR: 0.78, 95% CI: 0.58–1.04, per child when a 5-year lag-time was considered). Sibship size of the cases was also inversely associated with risk of testicular cancer (OR: 0.76, 95% CI: 0.66–0.88, per sibling). Age at first attempt to conceive was not associated with the risk of testicular cancer, and remarkably, there was no evidence of an association between our combined indicator of fertility (based on time to conception and use of assisted reproduction techniques) and risk of testicular cancer.

**Table 2:** Association between indicators of fertility and risk of testicular cancer.

	N° of cases (%)	N° of controls (%)	OR1^a^	95% CI^a^	OR2^a^	95% CI^a^
**Number of children 1 year before diagnosis/reference date**						
0	146 (63.8)	242 (59.5)	1.00	Ref	1.00	Ref
1	47 (20.5)	77 (18.9)	1.05	0.66-1-67	1.10	0.68–1.77
≥2	36 (15.7)	88 (21.6)	0.74	0.43–1.27	0.79	0.46–1.37
Missing	2	6				
Unit increase			0.85	0.66–1.09	0.88	0.68–1.13
**Number of children 5 years before diagnosis/reference date**						
0	169 (73.8)	274 (67.3)	1.00	Ref	1.00	Ref
1	36 (15.7)	64 (15.7)	0.91	0.54–1.52	0.95	0.56–1.61
≥2	24 (10.5)	69 (17.0)	0.56	0.30–1.04	0.63	0.33–1.18
Missing	2	6				
Unit increase			0.74	0.55–0.98	0.78	0.58–1.04
**Age at first attempt to conceive (years)**^**b**^						
<25	11 (19.0)	18 (18.4)	0.88	0.35–2.21	0.89	0.35–2.22
25–29	29 (50.0)	43 (43.9)	1.00	Ref	1.00	Ref
30–34	15 (25.9)	33 (33.7)	0.63	0.28–1.43	0.54	0.23–1.26
≥35	3 (5.2)	4 (4.1)	0.84	0.16–4.40	0.86	0.16–4.53
Unit increase			0.98	0.89–1.06	0.97	0.89–1.06
**Combined indicator of fertility (1 year before diagnosis/reference date)**						
Had children without attempting to conceive or attempting for less than 12 months	71 (31.6)	146 (37.0)	1.00	Ref	1.00	Ref
Did not have children and did not attempt to conceive	135 (60.0)	220 (55.7)	1.11	0.71–1.73	1.06	0.68–1.67
Attempted to conceive for at least 12 months and had or had not children, or had children using assisted reproduction techniques	19 (8.4)	29 (7.3)	1.28	0.67–2.45	1.04	0.53–2.05
Missing	6	18				
**Sibship size**						
1	59 (25.8)	74 (18.2)	1.00	Ref	1.00	Ref
2	105 (45.8)	174 (42.9)	0.71	0.46–1.09	0.72	0.46–1.11
≥3	65 (28.4)	158 (38.9)	0.52	0.33–0.83	0.54	0.34–0.86
Missing	2	7				
Unit increase			0.75	0.65–0.87	0.76	0.66–0.88

^a^ Adjusted for year of birth, residence (city of Turin, rest of the Province of Turin), method of recruitment (General Practitioners or hospital), age at diagnosis or reference age for controls, and educational level (junior high school, high school, university degree)

OR2 adjusted as OR1 and for cryptorchidism

^b^ Restricted to cases and controls who tried to have children at least 5 years before diagnosis or reference age for controls

OR, odds ratio; CI, confidence interval; Ref, reference

14 cases and 15 controls reported having visited a doctor for infertility problems up to 1 year before diagnosis/recruitment. After adjustment for cryptorchidism this rendered an OR of 1.32 (95% CI: 0.59–2.93), which increased to 1.88 (95% CI: 0.35–7.31) when the exposure was restricted to those reporting a physician-assigned diagnosis of infertility (11 cases and 11 controls) (data not shown). Adjustment for KITLG genotype did not substantially change these estimates (data not shown).

Restriction to patients without cryptorchidism and to patients diagnosed after 35 years of age altered the findings only marginally (data not shown). There was no substantial heterogeneity between the two histological subtypes, although the statistical power of the estimates was quite modest. Only when we considered the combined indicator of fertility and age at first attempt to conceive we observed different estimates and an opposite effect in the two histological subtypes; however the statistical power of these results was too weak to draw clear conclusions (See Supporting information, S1 and S2 Tables). We also tested the p-value for interaction between fertility indicators and histological type and we did not find evidence of heterogeneity between seminoma and nonseminoma (See Supporting information, S3 Table).

Among subjects with saliva samples, genotyping of rs995030 was successful in 202 cases and 333 controls (genotyping success rate: 98.7%); there was no evidence of departure from HWE (p = 0.10) among controls. Homozygous status for the risk allele (CC) was associated with an OR of testicular cancer of 5.38 (95% CI 1.21–23.91), compared with the homozygous status for the non-risk minor allele (TT). Additive analyses revealed an OR of 1.83 (95% CI 1.26–2.64) per 1-copy increase of the C allele (Table 3).

**Table 3:** Association between the KITLG SNP rs995030 and testicular cancer.

Genetic model	Genotype or Allele	Cases (N = 202) (%)	Controls (N = 342) (%)	OR^a^	95% CI^a^
**Codominant**	TT	2 (1.0)	15 (4.5)	1.00	Ref
	CT	39 (19.3)	91 (27.3)	3.22	0.70–14.80
	CC	161 (79.7)	227 (68.2)	5.38	1.21–23.91
	Missing	0	9		
**Additive (per allele)**	C			1.83	1.26–2.64

^a^ Adjusted for year of birth, residence (city of Turin, rest of the Province of Turin), and method of recruitment

OR, odds ratio; CI, confidence interval; Ref, reference

The association between number of children fathered before diagnosis/reference age and risk of testicular cancer was not modified by KITLG genotype, categorised as CC vs. TT/CT (Table 4). Similarly, no evidence of effect modification was found for the combined indicator of fertility status (data not shown). Last, we performed the analyses, restricted to control subjects, on the association between the KITLG genotype and the measures of subfertility collected. We did not find evidence of an association for number of children fathered 1 year and 5 year before diagnosis/reference date, age at first attempt of having children and for sibship size, although an indication of an association between KITLG genotype (CC vs CT/TT) and attempting to conceive for at least 12 months or fathering a child using assisted reproduction techniques, compared with attempting to conceive for less than 12 months before having a child, could be noted (unadjusted OR: 2.75, 95% CI: 0.89–8.53) (data not shown in Tables).

**Table 4:** Assessment of effect modification of KITLG on number of children fathered.

	Number of children 5 years before diagnosis	
Genotype	OR^a^	95% CI^a^
CT/TT	0.76	0.46–1.26
CC	0.74	0.53–1.02

^a^ Adjusted for year of birth, residence (city of Turin, rest of the Province of Turin), method of recruitment

(GPs or hospital), age at diagnosis or reference age for controls, educational level (junior high school, high school, university degree), and cryptorchidism.

OR, odds ratio, CI, confidence interval.

## Discussion

We used data from the EPSAM case-control study to explore the relation between subfertility and the risk of testicular cancer using questionnaire information on fecundity (i.e. number of children) and a combined indicator of fertility. We found an inverse association between the number of children fathered before cancer diagnosis and risk of testicular cancer, which was not explained by age at first attempt to conceive, as this variable was similar among cases and controls. Sibship size was inversely associated with the risk of testicular cancer. Conversely, we did not find an association between our combined indicator of fertility and risk of testicular cancer, although this indicator took into account at the same duration of attempt to conceive, unexpected pregnancies, and use of assisted reproduction techniques.

The association between fertility and testicular cancer has been investigated in a large number of studies. Number of children has consistently been reported to be associated with a decreased risk of testicular cancer both in questionnaire-based case-control studies [8–10] and in registry-based studies [5–7]. This association typically remains when analyses take into account socioeconomic factors or history of cryptorchidism, as in our study. Sibship size has also been repeatedly associated with a decreased risk of testicular cancer [11, 13].

Although our study is consistent with previous studies concerning number of children, we found no association between a combined indicator of fertility and risk of testicular cancer. If subfertility was actually associated with the development of testicular cancer, we would have expected to find a longer duration of attempt to conceive and/or an increased use of assisted reproduction techniques among cases compared to controls, but this was not observed.

Overall results from case-control studies using self-reported indicators of subfertility other than fecundity provide a conflicting picture [9, 10, 49–52]. Firstly, these studies are strongly heterogeneous: they used different indicators of subfertility and different, if any, lag-times between the age at assessment of the indicator and cancer diagnosis, and only some of them presented estimates adjusted for cryptorchidism. Indeed, most studies found that fertility problems are more frequent in cases than controls, but the magnitude of the estimates varied strongly and it was often impossible to exclude the possibility that reverse causation and/or confounding by cryptorchidism explained the reported effects.

Cohort studies with information on prospectively-obtained semen parameters have consistently found an association between impaired semen quality and risk of testicular cancer [14–19]. When the lag-time between semen analysis and cancer diagnosis was considered, the risk of testicular cancer seemed to be especially increased in the same year or in the year after semen analysis [5, 15], although it was also evident for longer lag times, indicating that the association was affected by reverse causation and/or detection bias to a certain extent. It is noteworthy that the magnitude of the associations was not dramatically large, and it is unclear if it could explain the inverse association between number of children fathered and risk of testicular cancer.

Overall, considering the evidence from register-based, case-control and cohort studies, it seems that the consistent, inverse associations between risk of testicular cancer and number of children and sibship size are not entirely paralleled or supported by the results of studies that tried to obtain more direct measurements of fertility.

Since heredity plays a strong role in testicular cancer, we performed genetic analyses to take this component into account. We did not test our samples for the gr/gr microdeletion on the Y chromosome, which is largely associated with impaired spermatogenesis [53–55] and the risk of testicular tumors [56] because it is a rare, low-penetrance allele (carrier frequency 2–3%) and thus accounts for a limited number of cases. The rs995030 SNP of the KITLG gene, which has been highly and consistently associated with risk of testicular cancer [21, 22, 45, 57] and is in strong linkage disequilibrium (r2>0.8) with other susceptibility SNPs [20, 22, 57] was selected for the present analyses.

Our results give further support to the existence of a strong link between KITLG and the risk of testicular cancer, but the variant that we analyzed did not modify the association between the number of children and the risk of testicular cancer, thus sustaining the conclusion of a previous study, which was based on a different indicator of male fertility, i.e. total sperm count [45].

Although our study has the strength of using multiple indicators to assess fertility, it has also some relevant limitations. First, our response rate was low, even if it was in line with most previous studies on testicular cancer. It should be noted, however, that our results on well-recognized risk factors for testicular cancer (including cryptorchidism and sibship size), are consistent with the literature. Although it is possible that non-response was driven particularly by fertility status, the lack of evidence of non-response bias for recognized risk factors is reassuring. Second, our analyses are based on self-reported information, with no clinical data available to confirm fertility status. However, this is unavoidable in case-control studies when a lag-time between fertility assessment and the diagnosis of testicular cancer is considered. Even in cohort studies the prospective collection of semen samples is feasible only in specific subgroups of the population (e.g. subjects with fertility problems, semen donors, etc…). Furthermore, the self-reported number of children fathered by a man is a crude indicator of fertility status. However, we think that any misclassification that could have been introduced by this indicator would be non-differential between cases and controls. Finally, some of the associations in our study had large CIs; thus, although we can draw conclusions as to the direction of the associations and the overall consistency, inference based on the single estimates should be carried out with caution.

## Conclusions

Our study supports previously reported inverse associations between testicular cancer and number of children and sibship size. However, our findings did not reveal an association between duration of attempt to conceive or use of assisted reproduction techniques and subsequent incidence of testicular cancer. The role of infertility, and the involved mechanisms, in testicular carcinogenesis remains to be elucidated.

## Supporting Information

S1 Table Association between indicators of fertility and risk of testicular cancer (restricted to nonseminoma subtype). Only cases with nonseminoma subtype and corresponding control subjects were included.
(PDF)

S2 Table Association between indicators of fertility and risk of testicular cancer (restricted to seminoma subtype). Only cases with seminoma subtype and corresponding control subjects were included.
(PDF)

S3 Table p-value for interaction between indicators of fertility and histological type (seminomas vs. nonseminomas) by comparing models with and without interaction by likelihood ratio test. (PDF)
