# Supplementary material for: Subfertility and Risk of Testicular Cancer in the EPSAM Case-Control Study
Source: PLoS One. 2016 Dec 30;11(12):e0169174. doi: 10.1371/journal.pone.0169174 (PMC5201268; doi:10.1371/journal.pone.0169174)
Supplement: S2 Table — Only cases with seminoma subtype and corresponding control subjects were included. (PDF) [file pone.0169174.s002.pdf]

S2 Table. Association between indicators of fertility and risk of testicular cancer (restricted to seminoma subtype)

	N° of cases (%)	N° of controls (%)	OR1[a]	95% CI[a]	OR2[a]	95% CI[a]
**Number of children 1 year before diagnosis/reference date**						
0	65 (51.2)	104 (49.1)	1.00	Ref	1.00	Ref
1	32 (25.2)	50 (23.6)	1.10	0.60-2.02	1.15	0.62-2.13
≥2	30 (23.6)	58 (27.4)	0.93	0.47-1.83	1.00	0.50-1.99
Missing	1	4				
Unit increase			0.93	0.67-1.27	0.96	0.70-1.32
**Number of children 5 year before diagnosis/reference date**						
0	79 (62.2)	121 (57.1)	1.00	Ref	1.00	Ref
1	27 (21.3)	42 (19.8)	1.00	0.52-1.93	1.06	0.55-2.06
≥2	21 (16.5)	49 (23.1)	0.67	0.32-1.42	0.76	0.35-1.63
Missing	1	4				
Unit increase			0.79	0.55-1.13	0.84	0.58-1.20
**Age at first attempt to conceive (years)[b]**						
<25	7 (16.3)	15 (21.7)	0.50	0.17-1.48	0.53	0.18-1.57
25-29	25 (58.1)	28 (40.6)	1.00	Ref	1.00	Ref
30-34	9 (20.9)	23 (33.3)	0.36	0.13-0.99	0.21	0.06-0.70
35+	2 (4.6)	3 (4.3)	0.54	0.07-3.97	0.54	0.07-4.03
Unit increase			0.96	0.87-1.07	0.94	0.85-1.05
**Combined indicator of fertility (1 year before diagnosis/reference date)**						
Had children without attempting to conceive or attempting for less than 12 months	54 (43.2)	92 (44.4)	1.00	Ref	1.00	Ref
Did not have children and did not attempt to conceive	59 (47.2)	94 (45.4)	0.97	0.54-1.74	0.92	0.51-1.65
Attempted to conceive for at least 12 months and had or had not children, or had children using assisted reproduction techniques	12 (9.6)	21 (10.1)	0.95	0.43-2.10	0.72	0.31-1.67
Missing	3	9				
**Sibship size**						
1	33 (26.2)	37 (17.3)	1.00	Ref	1.00	Ref
2	54 (42.9)	92 (43.0)	0.59	0.32-1.07	0.57	0.31-1.04
3+	39 (30.9)	85 (39.7)	0.51	0.27-0.94	0.51	0.28-0.96
Missing	2	2				
Unit increase			0.75	0.62-0.92	0.76	0.62-0.93

[a] Adjusted for year of birth, residence (city of Turin, rest of the Province of Turin), method of recruitment (General Practitioners or hospital), age at diagnosis or reference age for controls, and educational level (junior high school, high school, university degree)
OR2 adjusted as OR1 and for cryptorchidism
[b] Restricted to cases and controls who tried to have children at least 5 years before diagnosis or reference age for controls
OR, odds ratio; CI, confidence interval; Ref, reference
