# Supplementary material for: Subfertility and Risk of Testicular Cancer in the EPSAM Case-Control Study
Source: PLoS One. 2016 Dec 30;11(12):e0169174. doi: 10.1371/journal.pone.0169174 (PMC5201268; doi:10.1371/journal.pone.0169174)
Supplement: S3 Table — (PDF) [file pone.0169174.s003.pdf]

S3 Table. p-value for interaction between indicators of fertility and histological type (seminomas vs. nonseminomas) by comparing models with and without interaction by likelihood ratio test

	p-value
**Number of children 1 year before diagnosis/reference date**	0.11
**Number of children 5 year before diagnosis/reference date**	0.18
**Age at first attempt to conceive (years)[a]**	0.09
**Combined indicator of fertility (1 year before diagnosis/reference date)**	0.08
**Sibship size**	0.74

[a] Restricted to cases and controls who tried to have children at least 5 years before diagnosis or reference age for controls
